# Biologically Informed Treatment Approaches Toward Personalized Therapeutic Strategies in Lung Cancer

**DOI:** 10.3390/ijms27062855

**Published:** 2026-03-21

**Authors:** Kostas A. Papavassiliou, Amalia A. Sofianidi, Angeliki Margoni, Athanasios G. Papavassiliou

**Affiliations:** 1 First University Department of Respiratory Medicine, ‘Sotiria’ Chest Hospital, Medical School, National and Kapodistrian University of Athens, 11527 Athens, Greece; 2 Department of Biological Chemistry, Medical School, National and Kapodistrian University of Athens, 11527 Athens, Greece; amaliasof@med.uoa.gr (A.A.S.); mmargoni@med.uoa.gr (A.M.)

The therapeutic landscape of lung cancer treatment is moving from a one-size-fits-all approach to a more personalized one. Precision medicine, also referred to as personalized or individualized medicine, is an innovative approach that uses information about an individual’s genomic, environmental, and lifestyle data to guide medical decisions related to prevention, diagnosis, and treatment of diseases [1]. Indeed, lung cancer therapeutics represents one of the most thriving applications of precision medicine; actionable oncogenic drivers for approved first-line therapy include epidermal growth factor receptor (EGFR) mutations, anaplastic lymphoma kinase (ALK) fusions, c-ros oncogene 1 receptor tyrosine kinase (ROS1) rearrangements, v-raf murine sarcoma viral oncogene homolog B1 V600E (BRAF V600E) mutations, neurotrophic tyrosine receptor kinase (NTRK) rearrangements, rearranged during transfection (RET) rearrangements, Kirsten rat sarcoma viral oncogene homolog G12C (KRAS G12C) mutations, mesenchymal–epithelial transition factor (MET) alterations or amplifications, human epidermal growth factor receptor 2 (HER2) positivity, and neuregulin 1 (NRG1) gene fusions, introducing diverse treatment options for advanced non-small-cell lung cancer (NSCLC) [2]. However, precision oncology in lung cancer therapeutics moves several steps further: genomics, transcriptomics, epigenomics, proteomics, metabolomics, and immunology now direct treatment decisions. By identifying specific molecular drivers and immune biomarkers, clinicians can tailor therapies to each patient’s tumor profile more effectively and with less toxicity.

Unraveling the molecular features of the tumor is of outmost importance for the management of advanced NSCLC. Performing next-generation sequencing (NGS) in lung cancer tissue samples provides comprehensive genomic profiling [3]. Nevertheless, a tumor biopsy is not always feasible for every patient, and it can be accompanied by severe complications. Performing NGS in liquid biopsies is a novel approach towards personalized medicine. Molecular analysis of plasma-derived circulating tumor DNA (ctDNA) demonstrates comparable detection results to standard tissue analysis, with approximately 70% of patients having an equivalent outcome based on both tests [4]. Although this method cannot replace tissue testing, as its role is rather complementary [4], it offers novel insights into precision medicine, enabling real-time, dynamic monitoring of tumor evolution [5]. Liquid biopsy enables treatment escalation or de-escalation based on minimal residual disease (MRD) detection in each patient [6]. Beyond ctDNA, liquid biopsy offers perspectives into the exploration of circulating tumor cells, extracellular vesicles (EVs), tumor-educated platelets, and microRNAs (miRNAs) as pioneering biomarkers [7]. For example, elevated levels of plasma-derived miR-451a and miR-142-3p have been proposed as poor prognostic markers in NSCLC [8].

Artificial intelligence (AI) algorithms have been recently launched and continue to be developed, playing a central role in personalized medicine by enabling the integration and analysis of genomics, proteomics, clinical, and imaging datasets. A recently developed AI model can interpret liquid biopsy results and combine them with clinicopathological features, enhancing personalized clinical decision-making [9]. Different AI models are described in the literature, each using different biomarkers, but all toward the same goal: tailoring therapy to each patient and improving patient outcomes [10]. An aspiring AI model is APOLO11, which integrates clinical, radiologic, genetic, molecular, and immunological biomarkers of different lung cancer patients, aiming to predict therapy responses, resistance mechanisms, and biomarkers associated with increased therapy toxicity [11]. Notably, with regards to lung cancer immunotherapy, current biomarkers such as programmed death-ligand 1 (PD-L1) expression poorly predict immunotherapy outcomes [12]. Machine-learning algorithms hold the potential to address this gap. The Quantitative Vessel Tortuosity (QVT) score is a biologically interpretable imaging biomarker that quantifies tumor vascular complexity with radiomics. High QVT scores have been linked to poor immunotherapy outcomes, as disordered tumor vascularity promotes hypoxia and hinders immune evasion [13]. Other gene signatures associated with immunotherapy responses have been described with the use of AI, such as acyl-CoA synthetase medium chain family member 5 (ACSM5) [14] and DENN domain containing 1C (DENN1C) [15].

Precision medicine is tightly bound to biomarkers. A multicenter observational study conducted in Japan found CD8+ tumor-infiltrating lymphocyte (TIL) density to be a potential biomarker response in extensive-stage small-cell lung cancer (ES SCLC), facilitating enhanced patient selection for atezolizumab administration in combination with chemotherapy [16]. Another promising biomarker is the glucose-to-lymphocyte ratio (GLR), which correlates the metabolic profile of each patient to immune system function. An elevated GLR indicates metabolic and immune dysfunction, both reducing the efficacy of immunotherapy [17]. Similarly, exploiting metabolomics, the triglyceride glucose–body mass index (TyG-BMI) has been proposed as a biomarker of response to lung cancer immunotherapy; SCLC patients with a low TyG-BMI index are most likely to exhibit long-term survival benefit from chemoimmunotherapy combinations [18]. Biomarker-driven drug development is indisputably the future of oncology.

Delving deeper into the context of personalized medicine, the crucial role of transcription factors (TFs) and their cofactors should be highlighted. Several studies have underlined the role of TFs in predicting patient outcomes. For example, a three-gene signature based on tripartite motif containing 28 (TRIM28), interferon regulatory factor 3 (IRF3), and signal transducer and activator of transcription 3 (STAT3) demonstrated accuracy and sensitivity in predicting overall survival (OS) for patients with lung cancer [19]. Another area of interest is the exploitation of TFs for therapeutic use. As TFs are placed at the convergence of oncosignaling pathways and govern abnormal gene expression, hampering their function will probably result in the successful inhibition of tumor cell hallmarks without tumor cells being able to easily develop bypass mechanisms of resistance [20]. In this vein, several paradigms offering hopes and expectations based on clinical applicability come from other cancer types (e.g., direct targeting of TFs in certain leukemias, androgen- and estrogen-receptor modulators in prostate and breast cancer, indirect TF inhibition using cyclin-dependent kinase 4 and 6 (CDK4/6) inhibitors in breast cancer, hypoxia-inducible factor-2alpha (HIF-2α) inhibitors in Von Hippel–Lindau (VHL)-associated cancers, molecular glues and other degraders (or stabilizers of the ubiquitin ligase complex) against certain TFs such as Myc, nuclear factor erythroid 2-related factor 2 (NRF2)/Kelch-like ECH-associated protein 1 (KEAP1), and others). Nonetheless, various hurdles and limitations, such as a lack of well-defined small-molecule TF-binding pockets, reliance on complex DNA-TF, TF-TF or TF-cofactor interactions, high structural flexibility, demanding delivery/poor cell permeability, and low target selectivity, exist for this as a clinical approach in the real-world treatment of lung cancer so far. Prospective TF-targeting drugs may also be combined with other therapeutic agents, such as immune checkpoint inhibitors (ICIs), for the management of lung cancer in the clinical setting. One proposed approach is combining Janus kinase (JAK)/STAT inhibitors with PD-L1 blockade to foster immunotherapy efficacy [21].

Similarly, by analyzing the epigenetic profile of the tumor, treatment algorithms can be adjusted to each individual, addressing intra-patient heterogeneity [22]. Histone deacetylase (HDAC) inhibitors [23], DNA methyltransferases (DNMT) inhibitors [24], and several miRNA strategies [25] have been recently developed in lung cancer therapeutics. These strategies have also entered clinical trials. First-line pembrolizumab combined with the HDAC inhibitor vorinostat in patients with metastatic NSCLC was evaluated in a phase II randomized trial; however, this combination regimen did not perform as expected [26]. Likewise, the histone methyltransferase (HMT) inhibitor tazemetostat is under investigation in combination with topotecan and pembrolizumab in a phase I study in patients with recurrent SCLC [27]. The precise epigenetic landscape of lung cancer and the TF signatures associated with it have not yet been fully uncovered. Notwithstanding the limitations and challenges, bioinformatics and machine-learning approaches can identify transcription regulatory networks that control aberrant gene expression and map the underlying biological multifactorial conditions, elucidating the complexity and heterogeneity of this malignant disease [28].

In conclusion, precision medicine tailors medical care to each patient’s individual characteristics. Lung cancer treatment strategies are evolving as one of its most outstanding applications; biomarker-driven targeted therapies, immunotherapies, non-invasive liquid biopsy testing, and AI-guided algorithms that help unveil the profile and the behavior of the tumor are listed amongst them. Machine learning is undoubtedly the key to fulfill precision medicine, helping decipher TFs, epigenetic modulations, and other intricate molecular interactions. The future of lung cancer treatment lies in identifying the specific molecular profile of each lung tumor, while simultaneously attaining pharmacogenomic information to guide individualized therapy.

## Data Availability

Not applicable.
